# Multichromic Polymers Containing Alternating Bi(3-Methoxythiophene) and Triphenylamine Based Units with *Para*-Protective Substituents

**DOI:** 10.3390/ma9090779

**Published:** 2016-09-19

**Authors:** Yingfei Hou, Lingqian Kong, Xiuping Ju, Xiaoli Liu, Jinsheng Zhao, Qingshan Niu

**Affiliations:** 1 State Key Laboratory of Heavy Oil Processing, College of Chemical Engineering, China University of Petroleum (East China), Qingdao 266555, China; houyf@upc.edu.cn (Y.H.); qjniu@upc.edu.cn (Q.N.); 2 Dongchang College, Liaocheng University, Liaocheng 252059, China; lingqiankong@126.com (L.K.); jxp1127@163.com (X.J.); 3 Shandong Key Laboratory of Chemical Energy Storage and Novel Cell Technology, Liaocheng University, Liaocheng 252059, China; xiaoliliu1219@126.com

**Keywords:** 4-cyanotriphenylamine, 4-methoxytriphenylamine, bithiophenes derivatives, electrochromism, spectroelectrochemistry, electrochromic devices

## Abstract

Two novel triphenylamine-based thiophene derivative monomers, 4-cyano-4′,4″-di(4-methoxythiophen-2-yl)triphenylamine and 4-methoxy-4′,4″-di(4-methoxythiophen-2-yl)triphenylamine, were successfully synthesized. The corresponding polymers including poly (4-cyano-4′,4″-di(4-methoxythiophen-2-yl)triphenylamine) and poly (4-methoxy-4′,4″-di(4-methoxythiophen-2-yl)triphenylamine) were electrochemically synthesized and characterized by multiple test method. The electrochemical measurements and spectroelectrochemical analyses revealed that both of the two polymers had quasi-reversible redox behavior and multi-electrochromic properties. The two polymer films showed reversible electrochemical oxidation, excellent optical contrasts in NIR region (62% at 1070 nm for the first polymer, and 86% at 1255 nm for the second polymer), satisfactory coloration efficiencies and fast switching times. The research on the application of the as prepared polymer in the fabrication of electrochromic device was also conducted, employing PCMTPA or PMMTPA as the anodically coloring materials.

## 1. Introduction

The invention of conductive acetylene opens a new chapter in the basic and application research of conducting polymers (CP) [1]. Thereafter, they became the crucial materials in practical applications such as field effect transistors [2], photovoltaic [3], chemical sensors [4], thermal emission detectors, electrochromic materials [5], etc. Electrochromism was defined as the phenomenon that some materials displayed reversible optical change in absorption or transmittance when a doping–dedoping process was performed [6]. The CPs having electrochromism phenomenon, called electrochromic polymers (EC), have received enormous attention because they can change colors in a reciprocating way by altering their voltage potentials. The ECs polymers are usually prepared based on thiophene [7], pyrrole [8], fluorene [9], phenylene, or carbazole moieties [10], which have arisen people’s great concern owing to their fine-tunabilities of the band gaps (and the color), excellent processabilities, high optical contrasts, outstanding coloration efficiencies and low costs in comparison with a large number of inorganic oxides (nickel oxides, iridium and tungsten, etc.) materials [11,12,13,14,15]. Thus, most efforts of researchers in the electrochromic fields focused on design and synthesis of the multi-colored organic polymer with enhanced switching performance.

In recent years, TPA containing substances, especially related polymers, has attracted the interests of researchers worldwide, because of the versatile application of the materials in many fields, including hole transporters [16], electroluminescent materials [17], and electrochromic materials [18]. In addition to the excellent optical and electronic properties, TPA containing polymers are associated with some other valuable features, including amorphous microstructures, acceptable solubility, low glass transition temperatures, good film forming properties and so on [19]. High quality polytriphenylamine cannot be handily obtained by the electrochemical oxidation method since the formation of tetraphenylbenzidine as a byproduct, the undesirable dimer of TPA, is formed by tail to tail (para position) coupling during the anodic oxidation [20]. As a result of the presence of the dimer in polytriphenylamine, which usually show irreversible color changes during the dynamic electrochromic switches, polytriphenylamine (Poly(TPA)) cannot be used as ideal electrochromic materials [20]. The introduction of inactive substituent, e.g., methyl or cyano to one of the para-sites of TPA has been used for impeding the undesired side reactions, with the goal of promoting the formation of the linear, homogenous polymers, and finally enhancing the photoelectric properties of the polymers [21,22]. TPA-based conjugated polymers can be made by many methods. Based on the amino modification of the two para sites of the TPA derivatives, conjugated polyimides have been facilely obtained by the condensation polymerization reactions between diamines and aromatic dianhydrides, and the corresponding polyimides was subsequently available by thermal or chemical cyclodehydration method [23]. At the same time, TPA based polyazomethines have been prepared based on the condensation reactions between diamines (TPA containing) and aromatic dialdehydes [24]. At present, the Liou group have done much work on the TPA based polymers, including polyamides, polyimides and polyazomethines, most of which showed the multifunctional properties. Furthermore, as electrochromic materials, they usually showed P type doping process, and the colors of which were deepened from transmissive colorless state to coloring state as the potentials applied on the films increased gradually [21,22].

Polythiophenes and their derivatives represent another type of important conductive polymers, and usually possess excellent electrochromic properties, determined by their outstanding comprehensive performance, including tunable band gaps, reversible redox activities, low redox potentials, high ionic conductivity, as well as robust environmental stabilities [25,26,27]. It sounds like a good idea to introduce the TPA and thiophene units into the same polymer backbone, which is beneficial to take the advantages of two kinds of aromatic functional groups at the same time [28]. Recently, we reported the preparation of some of TPA-Thiophene hybrid monomers, derived from the substitute of the para-phenyl sites with thiophene moieties [29,30,31]. The TPA-Thiophene containing polymers have been achieved by the electrochemical oxidation of the monomers, which showed reversible color switches, fast switching time, and multichromic properties [29,30,31]. When one of the para-phenyl sites was substituted with inactive moieties (cyano or methoxyl), and two other sites are substituted with thiophene derivatives, the resulting monomers could be electrochemically oxidized to linear macromolecules with high electrochromic performance [29,31]. The polymerization reaction occurs at the *α* positions of the thiophene derivatives, and the band gaps of the obtained polymers were decreased due to the introduction of the thiophene derivatives to the poly(TPA)s, the stronger the electron donating abilities of the thiophene are, the lower the *E_g_* values of the generated polymers become [29]. More recently, 3-Methoxythiophene has been used for the construction of D-A type polymers as the donor moiety, and the resultant polymers were obtained with the similar *E_g_* values with that of the similar structured polymers when 3,4-Ethylenedioxythiophene (EDOT) was used as the donor unit, which suggested that 3-Methoxythiophene has a comparable electron donating ability to EDOT [32,33]. Furthermore, it has been shown that 3-Methoxythiophene has great potential in the preparation of high quality electrochromic materials, which might be attributed to its second advantage, i.e., the lower steric hindrance effect than that of the EDOT unit [33].

In this paper, 4-cyanotriphenylamine and 4-methoxytriphenylamine were synthesized beforehand, in which one of the phenyl groups was substituted by an electron-withdrawing cyano substituent or electron-donating methoxy substituent. Then, two Triphenylamine-Thiophene hybrid monomers, 4-cyano-4′,4″-di(4-methoxythiophen-2-yl)triphenylamine (CMTPA) and 4-methoxy-4′,4″-di(4-methoxythiophen-2-yl) triphenylamine (MMTPA), were synthesized by the conventional method. Through an electrodeposition approach, the corresponding polymers, PCMTPA and PMMTPA, could be formed and attached to the surface of the electrode. Subsequently, the electrochromic their properties are studied in many aspects, and the data obtained were used for a overall comparison of the two as prepared polymers with other structurally related polymers. The incorporating of the 3-Methoxythiophene unit in the main chain of the polymers extended the conjugation length, which reduced the band gaps of the polymers. Moreover, it was easily found that the band gaps of the polymers can be finely tuned by changing the substituent at para-position of TPA. Both polymers presented low oxidation potentials, high optical contrasts, high redox stabilities, high coloration efficiencies and extremely fast response times, which gave the polymers important application prospects in the field of smart electrochromism. The application of two kinds of conjugated polymers in the preparation of the electrochromic devices is also studied.

## 2. Experimental

### 2.1. Reagents and Equipment

Diphenylamine, Acetonitrile (ACN), dichloromethane (DCM), 3-methoxythiophene (99%), Poly (Methyl Methacrylate) (PMMA), LiClO_4_, and NaClO_4_ were obtained from Aladdin Reagent (Shanghai, China) Corp. Limited. Indium-tin-oxide-coated (ITO) glass (sheet resistance: <10 Ù·□^–1^) was obtained from Nanjing Chemical Reagent Co., Ltd. (Nanjing, China), and thoroughly cleaned before use. 4-fluoro-benzonitril, p-iodoanisole and Triglycol monomethyl ether (TEGDME) were obtained from Xiya Reagent (Chengdu, China). NaH, *N*,*N*-dimethyl formamide (DMF), CuI, K_2_CO_3_, Pd(PPh_3_)_2_Cl_2_ and Tetrahydrofuran were obtained from Tianjin Guangfu Chemical Corp. Limited (Tianjin, China) 2-(Tributylstannyl)-4-methoxyl-thiophene was prepared by the previous reported method. A Varian AMX 400 spectrometer was used for ^1^H NMR and ^13^C NMR measurement. SEM pictures were taken on a Hitachi S-4800 thermionic field emission SEM (Hitachi Ltd., Tokyo, Japan). Electrochemical measurements were performed by a CHI 760 C analyzer (CH Instrument, Shanghai, China). UV-vis-NIR spectra were recorded on a Varian Cary 5000 spectrophotometer (Agilent Ltd., Melbourne, Australia). Color photos of films and devices were taken using a commercial digital camera (Canon Ltd., Tokyo, Japan).

### 2.2. Synthesis of CMTPA and MMTPA

**Scheme 1:**
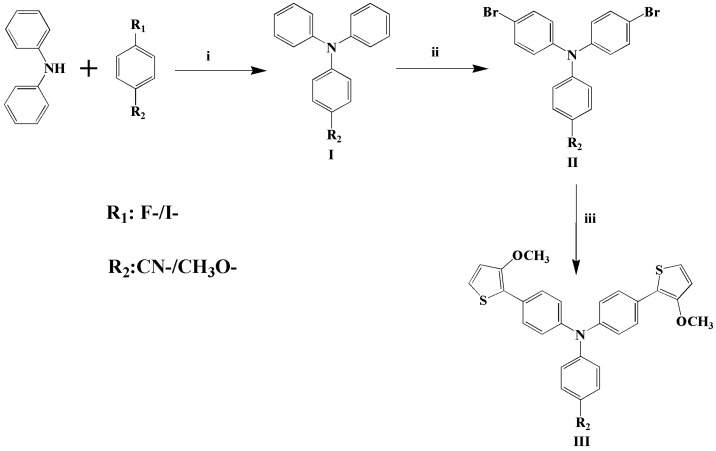
The synthesis procedure of the monomers: (**i**) NaH, DMF, reflux, 24 h; (**ii**) NBS, DMF, 0 °C, 12 h; and (**iii**) Pd(PPh_3_)_2_Cl_2_, dry THF, reflux, 24 h.

The syntheses of the 4-cyano-4′,4″-di(4-methoxythiophen-2-yl)triphenylamine (CMTPA) and 4-methoxy-4′,4″-di(4-methoxythiophen-2-yl) Triphenylamine (MMTPA) are depicted in Scheme 1. 4-cyanotriphenylamine and 4-methoxytriphenylamine were prepared according to previously reported method [29,31]. 4,4′-di(bromo)-4″-methoxytriphenylamine and 4,4′-di(bromo)-4″-cyanotriphenylamine were prepared through the bromine reaction of the above intermediates. The monomer including 4-cyano-4′,4″-di(4-methoxythiophen-2-yl)triphenylamine (CMTPA) and 4-methoxy-4′,4″-di(4-methoxythiophen-2-yl)triphenylamine (MMTPA) were synthesized by Stille cross coupling reaction with 2-(Tributylstannyl)(4-methyl-thiophene). The residue was purified by column chromatography on silica gel to give the target monomer CMTPA as yellow solid powder. ^1^H NMR (CDCl_3_, 400 M Hz, ppm): *δ* = 7.49 (dd, 4H, ArH), 7.13 (d, 4H, ArH), 7.06 (d, 2H, ArH), 6.94 (s, 2H, ArH), 6.21 (s, 2H, ArH). 3.94 (s, 6H). ^13^C NMR (CDCl_3_, *δ*, ppm): 158.89, 150.91, 146.80, 145.39, 131.53, 126.81, 126.17, 120.93, 119.68, 115.33, 103.69, 96.46, 57.50 (see Supplementary Materials Figure S1). Similarly, the target monomer MMTPA as yellow solid powder was achieved using Stille coupling reaction under the identical conditions. ^1^H NMR (CDCl_3_, 400 M Hz, ppm): *δ* = 7.41 (d, 4H, ArH), 7.10 (d, 2H, ArH), 7.30 (d, 4H, ArH), 6.87 (d, 4H, ArH), 6.14 (s, 2H), 3.82 (s, 9H). ^13^C NMR (CDCl_3_, *δ*, ppm): 158.10, 149.92, 145.22, 140.07, 130.18, 128.02, 127.19, 125.92, 124.21, 122.61, 120.66, 115.83, 97.63, 56.39 (see Supplementary Materials Figure S2).

### 2.3. Analytical Test Method

Electrochemistry: Electrodeposition, electrochemical analysis and spectroelectrochemical measurements were conducted as the methods previously reported by our group. The supporting solution used was 0.2 M NaClO_4_ dissolved in the mixed solvent ACN/DCM with the volume ratio of 1:1. For the electrosynthesis reactions taken by either CV or constant potential method, 5 mM of the relevant monomer was dissolved in the supporting electrolyte. The electrochemical characterization of the resultant polymers was performed directly in the electrolyte. In addition, the potential of Ag wire was emendated as 0.03 V vs. SCE in the supporting electrolyte used in this work [31].

Spectroelectrochemistry: For the spectroelectrochemistry test, the ITO electrode instead of platinum wire electrode was used as the working electrode (WE). The polymer films were potentially deposited on the ITO surface with a constant charge of 2.0 × 10^−3^ C with the active area of 0.9 cm × 2 cm. The three electrode system was constructed in a colorimetric cuvette with a fixed volume of 1 cm × 1 cm × 4.5 cm.

The preparation of electrochromic device (ECD): The gel electrolyte was prepared according to the previous reports. The sandwich type ECDs were prepared as our previous reports [30]. The as prepared PCMTPA (or PMMTPA) polymer was used as the anode, the most commonly used PEDOT was used as the cathode, and the gel electrolyte was used for the separation of two electrodes. The polymer films were deposited potentially on ITO glass electrodes with the active area of 1.8 cm ×1.6 cm. The polymerization potentials of the polymers are 1.1, 1.2 and 1.4 V vs. Ag wire, respectively, and the polymerization charge for all polymers was 3.6 × 10^−3^ C. The PCMTPA (or PMMTPA) film was fully dedoped before use, whereas the PEDOT film was in its doped state. In this way, the construction of ECD was accomplished.

## 3. Results and Discussion

### 3.1. Electrochemical Polymerization

**Scheme 2:**
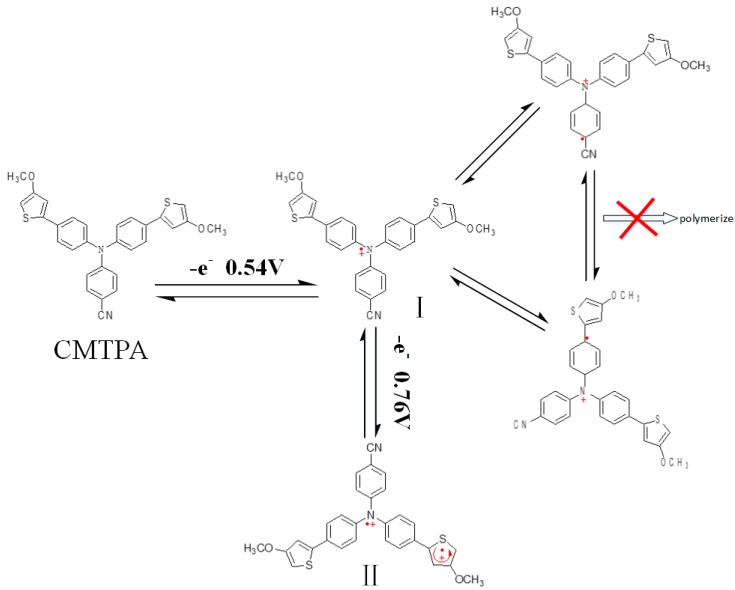
The coupling mechanism of CMTPA monomer.

**Figure 1:**
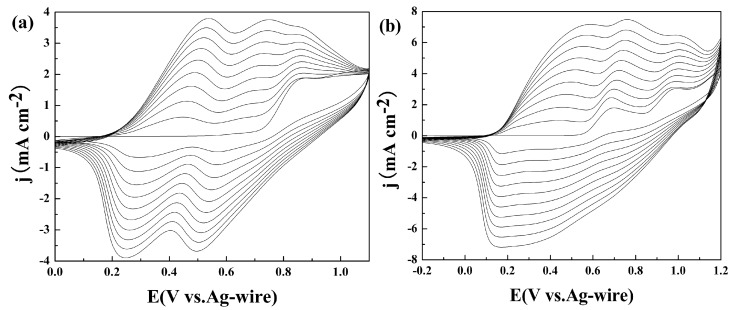
Repeated potential scanning of CMTPA (**a**) and MMTPA (**b**) in 0.2 M NaClO_4_/ACN/DCM solutions with a scan rate of 100 mV·s^−1^.

The polymerization behaviors of the two monomers are studied by CV method. The CV was conducted in the electrolyte containing 0.005 M of each monomer. The potential intervals were 0 V to 1 V for CMTPA, and −0.2 V to 1.2 V for MMTPA. The successive voltammetry curves of CMTPA and MMTPA are illustrated in Figure 1. The first cycle of CV of the monomers were related to their electrochemical oxidation. In Figure 1a, the onset oxidation potential (*E_onset_*) of CMTPA was observed at 0.58 V. The CV curves of CMTPA exhibited two coupled redox waves at 0.25–0.54 V, and 0.50–0.76 V. The first redox wave of CMTPA might be correlated with the production of the amine radical cationS (I), as shown in Scheme 2, which was stabilized by the presence of the electron-withdrawing cyano substituent or the electron-enriched methoxythiophene group at the para-position of TPA [31]. As a result, no coupling reactions occurred at this stage, which was confirmed by the absence of oligomers or polymers at the electrode. The second redox wave of CMTPA was caused by the oxidation of thiophene; that is to say, the production of reactive radical cations as reaction intermediates, and then the oxidative coupling at *α*-*α′* coupling position being achieved so that many oligomers appeared at the electrode (Scheme 2). As the scan was carried out, a deep colored substance was produced at the platinum wire, and the colors of the substance varied at the different potentials. Meanwhile, the increase of redox wave current intensities can also indicate that the amount of polymers formed on the wire increased [17].

The electrochemical deposition behavior of the monomer MMTPA was similar to that of the CMTPA, except the Eoneset potential of CMTPA was less than that of MMTPA. The general electro-oxidative polymerization mechanism of PMMTPA is the same as that of the PCMTPA (see Supplementary Materials Scheme S1). Similarly, as the cyclic scan continued, the insoluble colored substance was observed on the WE. Because of the difference in the substituent unit on the para-position of the triphenylamine unit, the shape of the CV curves of the monomers are different from each other to some extent, which suggested that the electrochemical properties of the polymers could be altered by the substituent effect.

### 3.2. Redox Characterization of the Polymer

**Figure 2:**
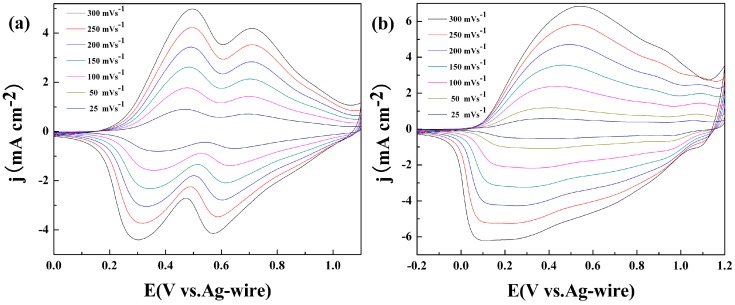

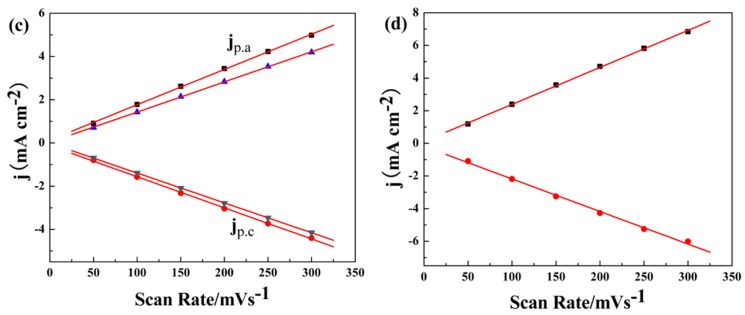
CV curves of the PCMTPA (**a**) and PMMTPA (**b**) films in the monomer-free electrolytes at different scan rates (**c**); and the relationships between anodic and cathodic peak current densities vs. scan rate for PCMTPA and PMMTPA, respectively (**d**).

The polymer films were produced on the Pt wire by the CV method for three cycles. Figure 2a,b shows the CV curves of the PCMTPA and PMMTPA films at different scan rates in monomer free supporting electrolyte, respectively. The PCMTPA and PMMTPA films presented distinct but different redox peaks. Two couples of redox peaks situated at 0.52–0.29 V and 0.73–0.55 V were clearly observed in the CV curves of the PCMTPA, which was due to redox behaviors of the cyanotriphenylamine core and the methoxythiophene arms, respectively, whereas PMMTPA showed only one couple of redox peaks at 0.54 V (oxidation peak) and 0.10 V (reduction peak), which demonstrated that the oxidation of 4-methoxytriphenylamine core and the methoxythiophene arms occurred simultaneously because of their strong interactions. The potential difference of the oxidation and reduction peaks between PCMTPA and PMMTPA revealed the influence made by the changes of the substituent unit at the para-position of TPA. Figure 2c,d shows the good linear relationships between the maximum current densities and scan rates of the two polymers, which indicated that the redox reaction that occurred at the surface of the polymer was not controlled by the diffusion of counter ions, even at every low scan rates [34].

### 3.3. Morphology

**Figure 3:**
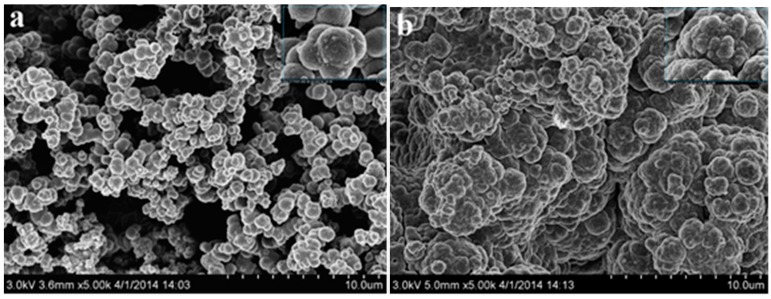
SEM images of PCMTPA (**a**) and PMMTPA (**b**) films deposited on the ITO electrode.

Figure 3 gives the SEM images of PCMTPA and PMMTPA films, which were made by constant potential method in the supporting electrolyte containing 0.005 M relevant monomers on ITO electrode. Both of the polymer films were dedoped before characterization. It is clearly observed that the two polymer films showed different surface and bulk morphologies, even though their structures are similar to some extent. As shown in Figure 3a, many small globules gathered together and formed the coral like structure on the surface of PCMTPA film and the mean diameter of the globules were about 1 µm. However, the PMMTPA reveals the accumulation clusters of cauliflowers with the dense arrangements uniformly dispersed on the surface of the film. Meanwhile, it is easily found that the diameter of the cauliflower is approximately 2 µm, which is much bigger than that of PCMTPA. The apparent difference in morphologies might be caused from the different polymerization rates, the higher current densities as presented in Figure 1. This phenomenon suggested the higher polymerization rates of PMMTPA than that of PCMTPA, which in turn formed a more condensed aggregation structure as shown in Figure 3b.

### 3.4. Optical Properties of the Monomers and Films

**Figure 4:**
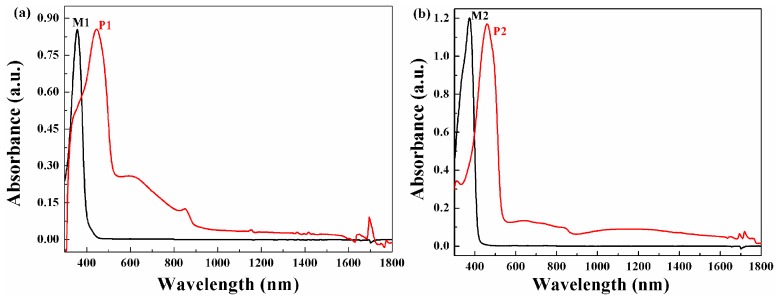
UV-vis absorption spectra of CMTPA and PCMTPA (**a**); and MMTPA and PMMTPA (**b**). Both CMTPA and MMTPA are dissolved in dichloromethane.

The UV-vis absorption spectra of CMTPA and MMTPA monomers dissolved in CH_2_Cl_2_ and the corresponding dedoped polymer films formed on ITO electrode were studied. The monomer CMTPA showed a main absorption peak (*λ_max_*) at 357 nm and exhibited an apparent red shift of 28 nm compared with that of the 4-cyanotriphenylamine unit (329 nm), which suggested that the introduction of methoxythiophene moieties to the two para-positions of the 4-cyanotriphenylamine extended the conjugation length of the molecules (Figure 4a) [35,36]. Similarly, MMTPA presented a maximum absorption peak located at 373 nm, which showed evident bathochromic shift of 70 nm by contrast with that of the 4-methoxytriphenylamine unit (303 nm) without thiophene derivatives conjugations (Figure 4b) [22]. The characteristic absorption of the monomer was due to the *π*-π* transitions. Meanwhile, it was clearly found that the *λ_max_* of MMTPA had a red shift of 16 nm compared to that of CMTPA, which was due to that strong electron-donating methoxy group on para-position of triphenylamine, and then increased the conjugation effect of MMTPA compound. The optical band gaps (*E_g_*) of CMTPA and MMTPA monomers were calculated as 3.12 eV and 3.01 eV, respectively, with the *E_g_* of MMTPA monomer was somewhat lower than that of CMTPA.

The UV-Vis profiles of the polymers prepared on ITO electrode in the reduced state were also presented in Figure 4. PCMTPA showed an absorption peak located at 445 nm (Figure 4a), while PMMTPA presented one absorption peak at 463 nm (Figure 4b). The maximum absorption peak of polymer PMMTPA exhibited a minor red shift compared with that of polymer PCMTPA. The *E_g_* of PCMTPA and PMMTPA polymers were 2.39 and 2.35, respectively.

It was found that both polymers showed noticeable shoulder peaks along side their main absorption peaks, with the peaks located at 593 nm and 636 nm for PCMTPA and PMMTPA, respectively, with the peak intensity of the former was much higher than that of the latter one (Figure 4). The shoulder peaks might be aroused by the intramolecular electron transfer (IET) from the 3-Methoxythiophene to the triphenylamine derivatives, which was absence for their respective monomers. As to PCMTPA, the presence of the cyano substituent on the para site of the triphenylamine unit will lead to the electron deficiency property of the triphenylamine core unit, which could promote the IET within the backbone, and then the higher peak intensity of the shoulder of PCMTPA than that of PMMTPA could be explained on this point.

**Scheme 3:**
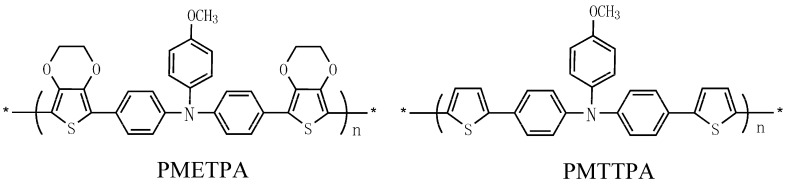
The molecular structures of PMETPA and PMTTPA.

Table 1 clearly lists the parameters of the two monomers, their corresponding polymers, and the previously reported structural analogs for comparison purpose. The molecular structures of PMETPA and PMTTPA are shown in Scheme 3. An apparent conclusion could be readily obtained: that the electron donating abilities of the thiophene derivatives as the para-site substituent have obvious effects on the conjugation length of the monomers and the corresponding polymers [29,31]. The stronger the electron donating abilities of the thiophene derivatives are, the higher the degrees of conjugation effect that can be anticipated. The order of the electron-donating capacities of the thiophene derivatives is as follows: 3,4-ethylenedioxythiophene > 3-Methoxythiophene > thiophene [29,31]. Besides, modification of the structure of the triphenylamine derivatives, as the intermediate linkage unit, could also be effective approaches for bandgap controlling of the polymers.

### 3.5. Spectroelectrochemistry

**Figure 5:**
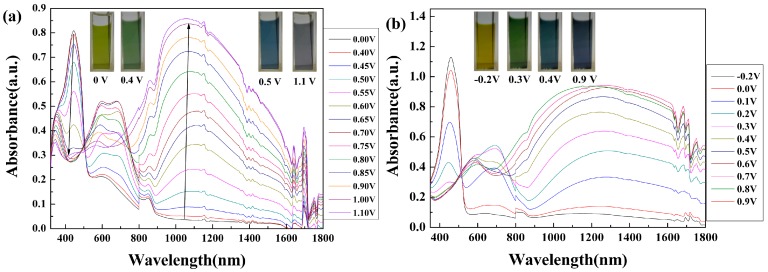

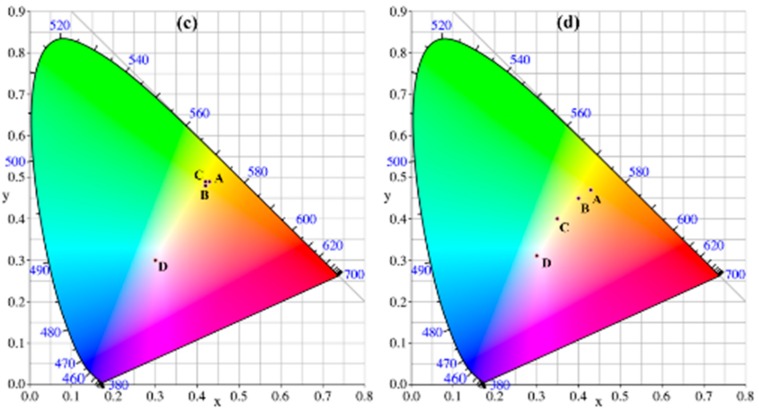
Uv-vis changes of film by the step by step oxidation of: PCMTPA film (**a**); and PMMTPA film (**b**); (**c**) Chromaticity diagram of PCMTPA film. The capital letters of A, B, C and D represented colors of film under 0 V, 0.4 V, 0.5 V and 1.1 V, respectively; (**d**) Chromaticity diagram of PMMTPA film. The capital letters of A, B, C and D represented colors of film under −0.2 V, 0.3 V, 0.4 V and 0.9 V, respectively.

The application of spectroelectrochemistry enables us to obtain information about the color changes and the production of polarons [35]. The changing absorption curves of the films were recorded upon the successive increased potentials applied on the polymers. Figure 5 presents the absorption curve of both PCMTPA and PMMTPA polymer films. As for the spectroelectrochemistry of PCMTPA polymer, the intensity of absorption peak centered at 445 nm and decreased uniformly as the film is oxidized gradually, while, at the same time, two new absorption peaks appeared at the longer wavelength, 700 nm and 1070 nm. Polarons were formed as the electrons escaped from the backbone of polymers in the course of p-doping. As shown in Figure 5a,c, the color of PCMTPA polymer film could vary among several colors: yellow green (0 V), green (0.4 V), blue (0.5 V), and dark blue (1.1 V). The multichromic properties of PCMTPA polymer gave it superior advantages in the application fields such as smart windows, photovoltaic devices and so on.

As can be seen in Figure 5b, the spectroelectrochemistry of PMMTPA polymer shows a *λ_max_* of 463 nm at 0 V (neutral state), which allows the transmittance of red and yellow light through the polymer film, and, since the eyes of people are more sensitive to yellow color than to red color, the PMMTPA film presents a yellow color. Two new peaks at 1185 nm and 1371 nm were formed synchronously due to appearance of polaron and bipolarons, with the increase of the oxidation depth. Furthermore, the peak at 463 nm decreased simultaneously with the increase of the potential differences applied on the PMMTPA film. Moreover, the PMMTPA polymer has a multi color characteristic: yellow color at 0 V, green color at 0.3 V, cyan color at 0.4 V, and blue color at 0.9 V. Moreover, the color changes of the polymers were more specifically characterized using CIE1976 color space (*L*, *a*, *b*) and are summarized in Table 2. The yellow, green and the blue colors belong to the primary colors, and the electrochromic polymers, which can switch between these primary colors, are considered an urgently-needed type of polymers. The contribution of the present work lies in these properties of PCMTPA and PMMTPA.

Although the newly synthetic polymers have similar backbones, the PCMTPA and PMMTPA polymer films showed different spectroelectrochemical properties and colors due to the difference in the molecular structure of the triphenylamine derivatives as the linkage unit. It is beneficial to improve the conjugation length of the polymers to enhance the electron donating abilities of the linkage units between the bithiophenes.

### 3.6. Electrochromic Switching of the Polymer Films in Solution

**Figure 6:**
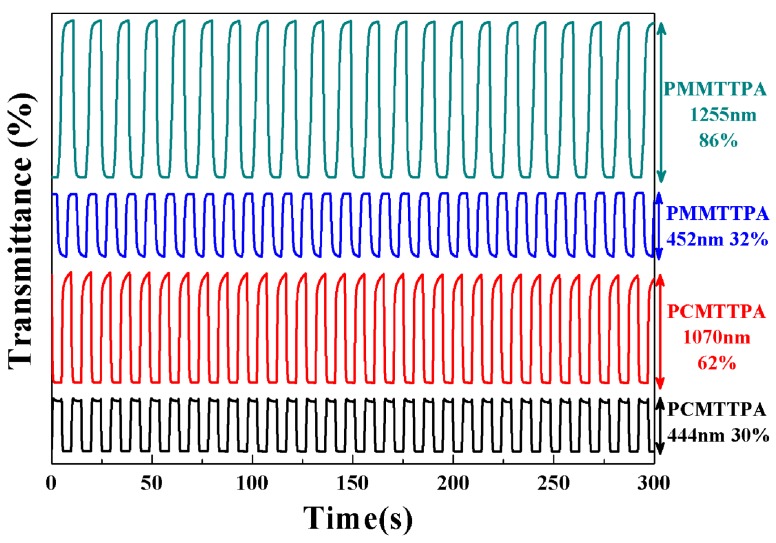
The dynamic switching graph of the polymers including PCMTTPA and PMMTTPA.

The switch dynamic of the polymer films is an important factor for determining the application aspect of materials, and is tested in monomer free electrolyte. The potential conversion of PCMTPA was made between 0 and 1.1 V, with the time interval of 5 s at the wavelength of 444 nm and 1070 nm. The applied potentials of PMMTPA varied between −0.2 and 0.9 V, and the detection wavelengths are at 500 nm and 1255 nm with the time intervals of 5 s and 7 s, respectively. In Figure 6, the switching properties of the two polymer films could be easily inferred.

There are three main parameters to evaluate the dynamic characteristics of the polymer films including optical contrast (*T%*), response time (*t*) and coloration efficiency (*CE*). For the physical meaning and calculation method of the above parameters, the reader is referred to previous reports [32]. The dynamic switching properties of PCMTPA and PMMTPA are presented in Table 3. Both of the polymers presented fast switching properties, great coloration efficiencies and especially high optical contrasts at near infrared (NI) region, which makes these polymers promising candidates as active materials for preparation of NI devices. Table 3 also enumerates the dynamic properties of previously reported polymers including PMTTPA and PMETPA, from which it can be seen that the two polymers reported here have comparable performance to the previously reported polymers in respect to *∆T%* and *t*, however, the *CE* of the newly reported polymers are inferior to the previous reported ones.

### 3.7. Spectroelectrochemical Properties of ECDs

**Figure 7:**
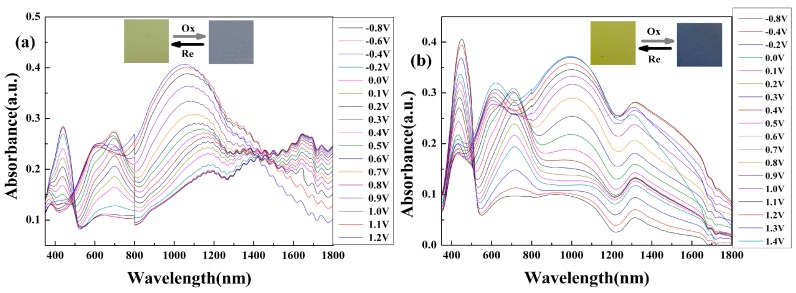
(**a**) Spectroelectrochemical spectra of PCMTPA/PEDOT device; and (**b**) PMMTPA/PEDOT device as the potentials applied increased.

PCMTPA or PMMTPA films prepared in this work were used as an anodically coloring polymer, and Poly (3,4-ethylenedioxythiophene) (PEDOT) was used as a cathodically coloring polymer; these were used as the anode and cathode, respectively, for the fabrication of electrochromic device (ECDs). The PCMTPA/PEDOT device was made by inserting the PMMA based solid electrolyte between PCMTPA film layer and PEDOT film layer, and the PMMTPA/PEDOT device was prepared in the same way. The PCMTPA/PEDOT device switched between yellow color (−0.8 V) and blue color (1.4), as shown in the inset of the Figure 7a. As shown in the electrospectrochemistry of the PCMTPA/PEDOT device (Figure 7a), during the gradual oxidation of the device, the peak at 440 nm decrease, and two new absorption peaks appeared in the visible (693 nm) and near infrared regions (1070 nm). Similarly, the PMMTPA/PEDOT device switched between bright yellow (−0.8 V) and deep blue color (1.4 V) colors.

**Figure 8:**
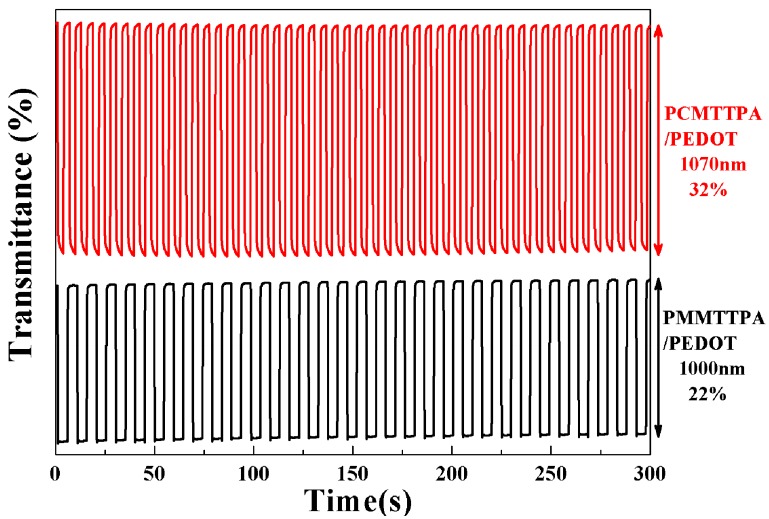
Electrochromic switching response for PCMTPA/PEDOT device at 1070 nm and PMMTPA/PEDOT device films monitored at 1000 nm, both at various potentials with the different residence times (3 s and 5 s).

Figure 8 shows the electrochromic switching properties of two devices, from which the specific parameters were calculated and be shown in Table 4. In addition, the corresponding properties of PMETPA/PEDOT and PMTTPA/PEDOT previously reported by our group are also exhibited in Table 4, from which it can be seen that the switching properties of the six ECDs are comparable to each other [29,31]. The ECDs constructed in this paper have better comprehensive performance, and are expected to be applied in industrial utilizations, including energy saving glass, upon the realization of the extended dimensions of the ECDs through the optimization of the fabrication technology.

### 3.8. Open Circuit Memory of ECDs

**Figure 9:**
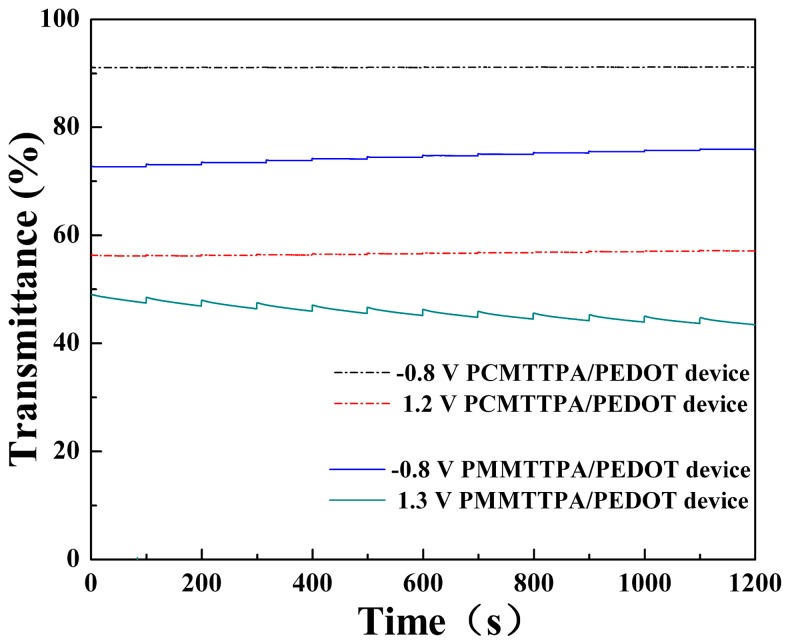
Open circuit memory of PCMTPA/PEDOT device monitored at 1070 nm, and PMMTPA/PEDOT device monitored at 1000 nm by single-wavelength absorption spectroscopy.

**Figure 10:**
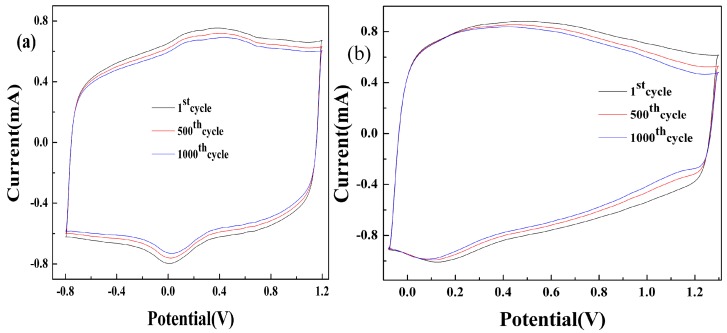
CV of both PCMTPA/PEDOT (**a**); and PMMTPA/PEDOT (**b**) devices as a function of repeat with the scan rate of 500 mV·s^–1^.

After applying a pulse potential of 1 s, the color retention property of the device is detected by the changes of absorption [21]. As shown in Figure 9, there were almost no transmittance change both in oxidized and reduced states at 1070 nm, which indicated that the PCMTPA/PEDOT device showed extreme stability and good optical memory. As for the PMMTPA/PEDOT device, its transmittance of the reduced state was somewhat stable at 1000 nm while the oxidized state exhibited a 5% loss. In this sense, the two devices showed good optical memory. The stability of the PCMTPA/PEDOT and PMMTPA/PEDOT devices were tested by the CV method. Figure 10 shows the changes of CV curves for both devices between the 1st and 500th/1000th cycles. From the calculation, it was found that 93.6% of its original CV area persisted after 500 cycles for PCMTPA/PEDOT. With the scan continuing, 90.4% of the original CV area was retained at the 1000th cycle. As for the PMMTPA/PEDOT device, 99.0% of its CV area was maintained after 500 cycles. After 1000 cycles, 96.5% of its original CV area was persisted. The PMMTPA/PEDOT device has a comparable stability with that of the PCMTPA/PEDOT device. Excellent environmental stability is required for industrial application, so these two devices have very good application prospects [30].

## 4. Conclusions

In summary, two novel monomers employing 4-cyanotriphenylamine or 4-methoxytriphenylamine unit as the linkage unit and bi(3-Methoxythiophene) as the substituent group were synthesized. The corresponding polymers are obtained by the electrosynthesis method, and are named PCMTPA and PMMTPA, respectively. Electrochemical and spectroelectrochemical measurements were undertaken for the characterization of the as prepared polymers. Both polymer films displayed multichromic properties and exhibited excellent optical contrasts in the NIR region (62% at 1070 nm for PCMTPA, and 86% at 1255 nm for PMMTPA), which makes the polymers good candidates for electrochromic display applications in NIR region. More importantly, the colors of both polymers could switch among more than two primary colors, which are regarded as an indispensible property for high-quality electrochromic materials. The changes in the pendent groups of the triphenylamine derivatives as the intermediate linkage units could also be used for the tuning of the bandgaps of the polymers. Furthermore, the made and tested dual-type ECDs employing PCMTPA or PMMTPA as their anodic materials were also conducted. The PCMTPA/PEDOT device switched between yellow and blue color, while the PMMTPA/PEDOT device offered a bright yellow color at neutral state and showed a dark blue color at oxidized state.

## Figures and Tables

**Table 1:** The experimental parameters including *E_onset_*, *λ_max_*, onset of the optical absorption spectra (*λ_onset_*), *E_g_* ^a^, HOMO/LUMO energy levels and the calculated data of the monomers from the Gaussian 03 programs.

Compounds	*E_onset_*, vs. (Ag-wire) (V)	*λ_max_* (nm)/*λ_onset_* (nm)	*E_g_* ^a^ (eV)	HOMO ^b^ (eV)	LUMO ^c^ (eV)
CMTPA	0.58	357/445	3.12	−5.01	−1.89
MMTPA	0.45	373/463	3.01	−4.88	−1.87
METPA ^d^	0.46	371/418	2.96	−4.86	−1.90
MTTPA ^d^	0.49	363/415	2.98	−4.89	−1.91
PCMTPA	0.16	445/607	2.39	−4.59	−2.2
PMMTPA	0.09	463	2.35	−4.52	−2.17
PMETPA ^d^	0.11	454/533	2.32	−4.51	−2.19
PMTTPA ^d^	0.18	435/522	2.37	−4.58	−2.21

^a^ Calculated from the low energy absorption edges (*λ_onset_*), *E_g_* = 1241/*λ_onset_*; ^b^ HOMO = −e(*E_onset_* + 4.4); ^c^ Calculated by the addition of the optical band gap to the HOMO level; ^d^ Data were taken from Ref. [29].

**Table 2:** The values of *L*, *a*, *b* for both of PCMTPA and PMMTPA polymers in different states.

Polymers	*E*, vs. (Ag wire) (V)	*L*	*a*	*b*	Colors
PCMTPA	0	61.08	−9.50	56.56	yellow
	0.4	63.01	−8.60	62.15	yellowish green
	0.5	61.94	−11.31	57.42	steel blue
	1.1	53.76	4.98	−7.10	gray
PMMTPA	−0.2	82.58	−4.07	70.32	bright yellow
	0.3	78.71	−7.69	54.84	dark yellowish green
	0.4	72.69	−12.22	25.59	dark blue green color
	0.9	69.25	1.36	−6.67	dark blue

**Table 3:** The optical contrast (*∆T%*), response time (*t*), and coloration efficiency (*CE*) of PCMTPA and PMMTPA.

Compounds	Wavelength (nm)	*∆T*%	Response Time (*t*, s)	*CE* (cm^2^·C^−1^)
PCMTPA	444	30	0.8	112
	1070	62	4.5	124
PMMTPA	452	32	1.1	145
	1255	86	3.6	164
PMTTPA ^a^	430	28.7	0.9	177
	1280	55.5	0.4	305
PMETPA ^a^	454	43.7	1.3	230
	1200	77.6	1.47	201

^a^ Data were taken from Ref. [27].

**Table 4:** The optical contrast (*ΔT%*), response time, color and coloration efficiency (*CE*) of the PCMTPA/PEDOT and PMMTPA/PEDOT devices.

Compounds	Optical Contrast (*ΔT%*)	Response Time (s)	Color	Coloration Efficiency (cm^2^·C^−1^)
PCMTPA/PEDOT	32 (1070 nm)	0.6 (1070 nm)	yellow/blue	301 (1070 nm)
PMMTPA/PEDOT	22 (440 nm)	1.80 (440 nm)	green/blue	235 (440 nm)
PMETPA/PEDOT ^a^	29.8 (640 nm)	0.40 (640 nm)	yellowish green/blue	270 (640 nm)
PMTTPA/PEDOT ^a^	30.3 (600 nm)	1.80 (600 nm)	yellow/stone-blue	517 (600 nm)

^a^ Data were taken from Ref. [27].

## Supplementary Materials

The following are available online at www.mdpi.com/1996-1944/9/9/779/s1. Figure S1: (a) ^1^H NMR spectrum of 4-cyano-4′,4″-di(4-methoxythiophen-2-yl) triphenylamine (CMTPA) in CDCl_3_. Solvent peak is at *δ* = 7.26 ppm; (b) ^13^C NMR spectrum of 4-cyano-4′,4″-di(4-methoxythiophen-2-yl) triphenylamine (CMTPA) in CDCl_3_. Solvent peak is at *δ* = 77.3 ppm; Figure S2: (a) ^1^H NMR spectrum of 4-methoxy-4′,4″-di(4-methoxythiophen-2-yl) triphenylamine (MMTPA) in CDCl_3_. Solvent peak is at *δ* = 7.26 ppm; (b) ^13^C NMR spectrum of 4-methoxy-4′,4″-di(4-methoxythiophen-2-yl) triphenylamine (MMTPA) in CDCl_3_. Solvent peak is at *δ* = 77.3 ppm; Scheme S1: The coupling mechanism of MMTPA monomer.
