# Supplementary material for: Multichromic Polymers Containing Alternating Bi(3-Methoxythiophene) and Triphenylamine Based Units with Para-Protective Substituents
Source: Materials (Basel). 2016 Sep 19;9(9):779. doi: 10.3390/ma9090779 (PMC5457089; doi:10.3390/ma9090779)
Supplement: Supplementary file 1 [file materials-09-00779-s001.pdf]

Supplementary Materials: Multichromic Polymers Containing Alternating Bi(3-Methoxythiophene) and Triphenylamine Based Units with *Para*-Protective Substituents

Yingfei Hou, Lingqian Kong, Xiuping Ju, Xiaoli Liu, Jinsheng Zhao and Qingshan Niu

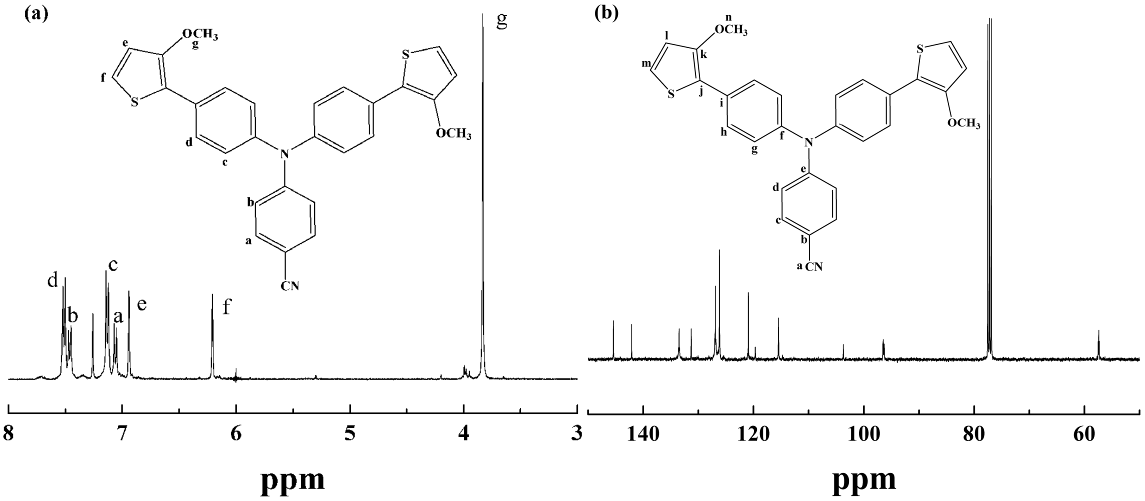

Figure S1. (**a**) ^{1}H NMR spectrum of 4-cyano-4′,4″-di(4-methoxythiophen-2-yl) triphenylamine (CMTPA) in $CDCl_3$. Solvent peak is at δ = 7.26 ppm; (**b**) ^{13}C NMR spectrum of 4-cyano-4′,4″-di(4-methoxythiophen-2-yl) triphenylamine (CMTPA) in $CDCl_3$. Solvent peak is at δ = 77.3 ppm.

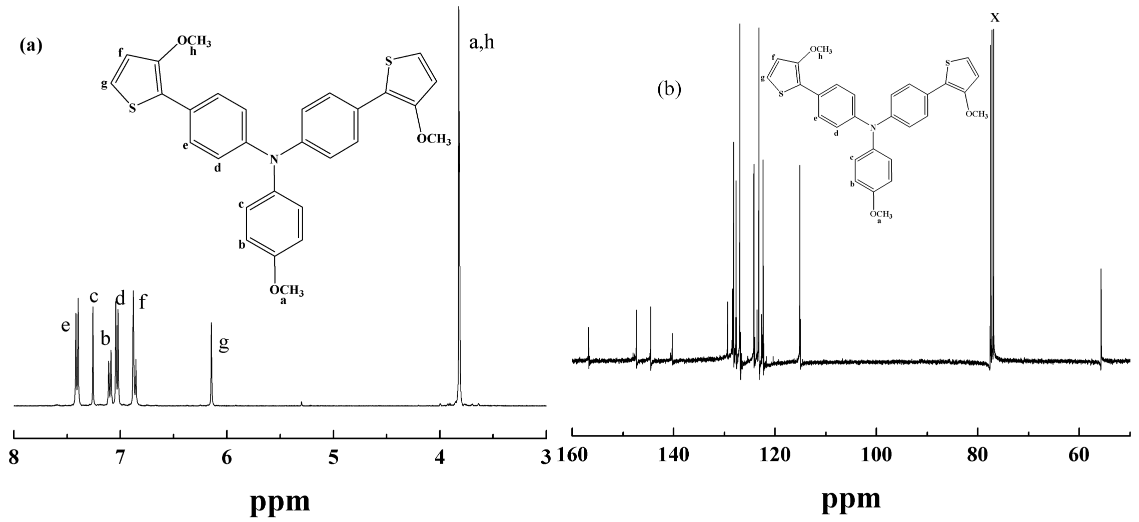

Figure S2. (**a**) ^{1}H NMR spectrum of 4-methoxy-4′,4″-di(4-methoxythiophen-2-yl) triphenylamine (MMTPA) in $CDCl_3$. Solvent peak is at δ = 7.26 ppm; (**b**) ^{13}C NMR spectrum of 4-methoxy-4′,4″-di(4-methoxythiophen-2-yl) triphenylamine (MMTPA) in $CDCl_3$. Solvent peak is at δ = 77.3 ppm.

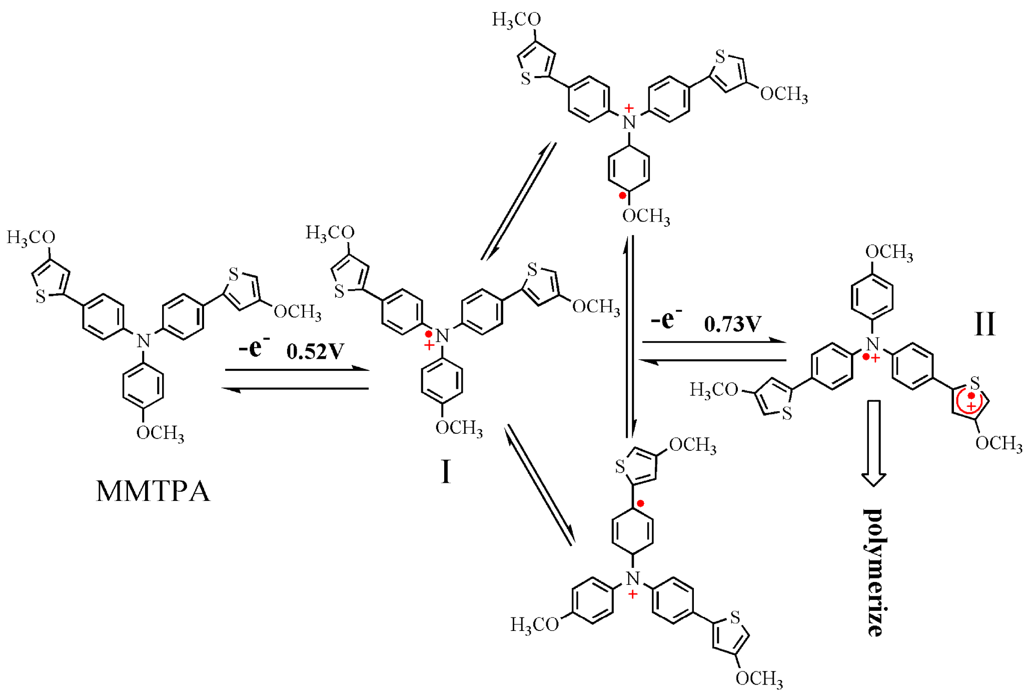

Scheme S1. The coupling mechanism of MMTPA monomer.
